# Analysis of the asymmetrically expressed *Ablim1 *locus reveals existence of a lateral plate Nodal-independent left sided signal and an early, left-right independent role for nodal flow

**DOI:** 10.1186/1471-213X-10-54

**Published:** 2010-05-20

**Authors:** Jonathan Stevens, Alexander Ermakov, Jose Braganca, Helen Hilton, Peter Underhill, Shoumo Bhattacharya, Nigel A Brown, Dominic P Norris

**Affiliations:** 1 MRC Mammalian Genetics Unit, MRC Harwell, Harwell Science and Innovation Campus, Oxfordshire, OX11 0RD, UK; 2 Dept of Cardiovascular Medicine, University of Oxford, Wellcome Trust Centre for Human Genetics, Roosevelt Drive Headington Oxford OX3 7BN, UK; 3 Division of Basic Medical Sciences, St George's University of London, Cranmer Terrace, London SW17 0RE, UK; 4 Centre for Regenerative Medicine Chancellor's Building, 49 Little France Crescent, Edinburgh EH16 4SB, UK; 5 José Bragança Departamento de Ciências Biomédicas e Medicina, Universidade do Algarve, Campus de Gambelas, 8005-139 Faro, Portugal; 6 IBB-Institute for Biotechnology and Bioengineering, Centro de Biomedicina Molecular e Estrutural, UAlg. Portugal

## Abstract

**Background:**

Vertebrates show clear asymmetry in left-right (L-R) patterning of their organs and associated vasculature. During mammalian development a cilia driven leftwards flow of liquid leads to the left-sided expression of *Nodal*, which in turn activates asymmetric expression of the transcription factor *Pitx2*. While *Pitx2 *asymmetry drives many aspects of asymmetric morphogenesis, it is clear from published data that additional asymmetrically expressed loci must exist.

**Results:**

A L-R expression screen identified the cytoskeletally-associated gene, actin binding lim protein 1 (*Ablim1*), as asymmetrically expressed in both the node and left lateral plate mesoderm (LPM). LPM expression closely mirrors that of *Nodal*. Significantly, *Ablim1 *LPM asymmetry was detected in the absence of detectable *Nodal*. In the node, *Ablim1 *was initially expressed symmetrically across the entire structure, resolving to give a peri-nodal ring at the headfold stage in a flow and *Pkd2*-dependent manner. The peri-nodal ring of *Ablim1 *expression became asymmetric by the mid-headfold stage, showing stronger right than left-sided expression. Node asymmetry became more apparent as development proceeded; expression retreated in an anticlockwise direction, disappearing first from the left anterior node. Indeed, at early somite stages *Ablim1 *shows a unique asymmetric expression pattern, in the left lateral plate and to the right side of the node.

**Conclusion:**

Left LPM *Ablim1 *is expressed in the absence of detectable LPM *Nodal*, clearly revealing existence of a *Pitx2 *and *Nodal*-independent left-sided signal in mammals. At the node, a previously unrecognised action of early nodal flow and Pkd2 activity, within the pit of the node, influences gene expression in a symmetric manner. Subsequent *Ablim1 *expression in the peri-nodal ring reveals a very early indication of L-R asymmetry. *Ablim1 *expression analysis at the node acts as an indicator of nodal flow. Together these results make *Ablim1 *a candidate for controlling aspects of L-R identity and patterning.

## Background

While vertebrates are externally mirror symmetrical between their left and right sides, internally the positioning and patterning of their organs and vasculature show marked left-right (L-R) asymmetry. The heart and its associated vasculature, the lungs and various elements of the gut show distinct L-R asymmetric patterning. The importance of correctly establishing L-R asymmetry is evident when the association between situs defects and disease is analysed. A strong association is evident with congenital heart disease [1] while links also exist with ciliary dyskinesia, cystic kidney disease and extrahepatic biliary atresia [2,3].

During mammalian development, the first morphological sign of L-R asymmetry is the looping of the primitive heart tube, initially to the right. Shortly after this the embryo begins to undergo embryonic turning, the process that results in the embryo taking up the classic foetal position. This occurs in a L-R asymmetric manner such that the caudal-most region of the embryo passes to the right side of the head. These morphological asymmetries are, however, prefigured by molecular asymmetries. Work over the past decade has resulted in a broadly accepted model explaining how L-R asymmetry is established in the mammalian embryo (reviewed [4]).

Initial asymmetry is believed to be established when posteriorly tilted cilia within the embryonic node rotate to drive a leftwards flow of liquid (nodal flow). The role of flow in establishing situs was demonstrated in elegant experiments applying artificial flow to embryos in culture; flow reproducibly directed normally left-sided gene expression downstream of the direction of flow [5]. The question of how the embryo perceives nodal flow remains unresolved, although various models exist. One model argues that a morphogen is carried leftwards by the flow [6]. A second, the two cilia model [7], argues that mechanosensory cilia directly sense nodal flow, resulting in a left-sided intracellular calcium signal. The third model argues that membrane bound vesicles, termed nodal vesicular parcels (NVPs), are carried leftwards by the flow, breaking on the left side of the node to release a cargo of morphogens [8]. At present no one model fully explains all the existing experimental data [9]. The resulting signal at the left side of the node is then communicated several cell diameters to the left lateral plate, possibly through intracellular calcium signalling [7].

In the left lateral plate, the gene encoding the signalling molecule Nodal is asymmetrically activated downstream of nodal flow. Little is known of the mechanism of this activation. *Nodal *is at the top of a left-sided genetic cascade, auto-activating its own expression as well as that of its antagonist *Lefty2 *and the downstream transcription factor *Pitx2 *(reviewed [4]). All are asymmetrically expressed prior to the appearance of morphological asymmetry [10]. While *Nodal *and *Lefty2 *are expressed for only 6-8 hours [10], asymmetric *Pitx2 *expression is maintained into organogenesis and has been argued to be the ultimate effecter of left identity [11,12]. However, *Pitx2 *null embryos do not lose all aspects of left sidedness, making it clear that additional uncharacterised signals help distinguish the left and right sides of the early embryo [4].

We set out to identify additional asymmetric genes using a micro array based approach. This resulted in the identification of asymmetric expression of actin binding lim protein 1 (*Ablim1*), a gene showing asymmetry of expression in both the left LPM and the node. The lateral plate expression broadly mirrors that of *Nodal*, yet uniquely for a left LPM expressed locus, we demonstrate that it can be asymmetrically expressed in the absence of detectable LPM *Nodal*. This makes it clear that there is an additional uncharacterised asymmetric LPM signal. Initial *Ablim1 *expression in the ventral node was downregulated and peri-nodal expression upregulated in a flow and *Pkd2*-dependent manner, revealing an asymmetry independent role for flow in regulating gene expression within the node. The first node asymmetry was seen at the late headfold stage, earlier than any previously characterised L-R asymmetry in the mouse. Subsequently, the peri-nodal ring of expression retreated asymmetrically, moving around the node in a clockwise direction. Together these results reveal a *Nodal*-independent asymmetric LPM signal and make *Ablim1 *a candidate for controlling aspects of L-R identity and patterning.

## Results

### *Ablim1*: a novel mammalian L-R asymmetric locus

**Figure 1:**
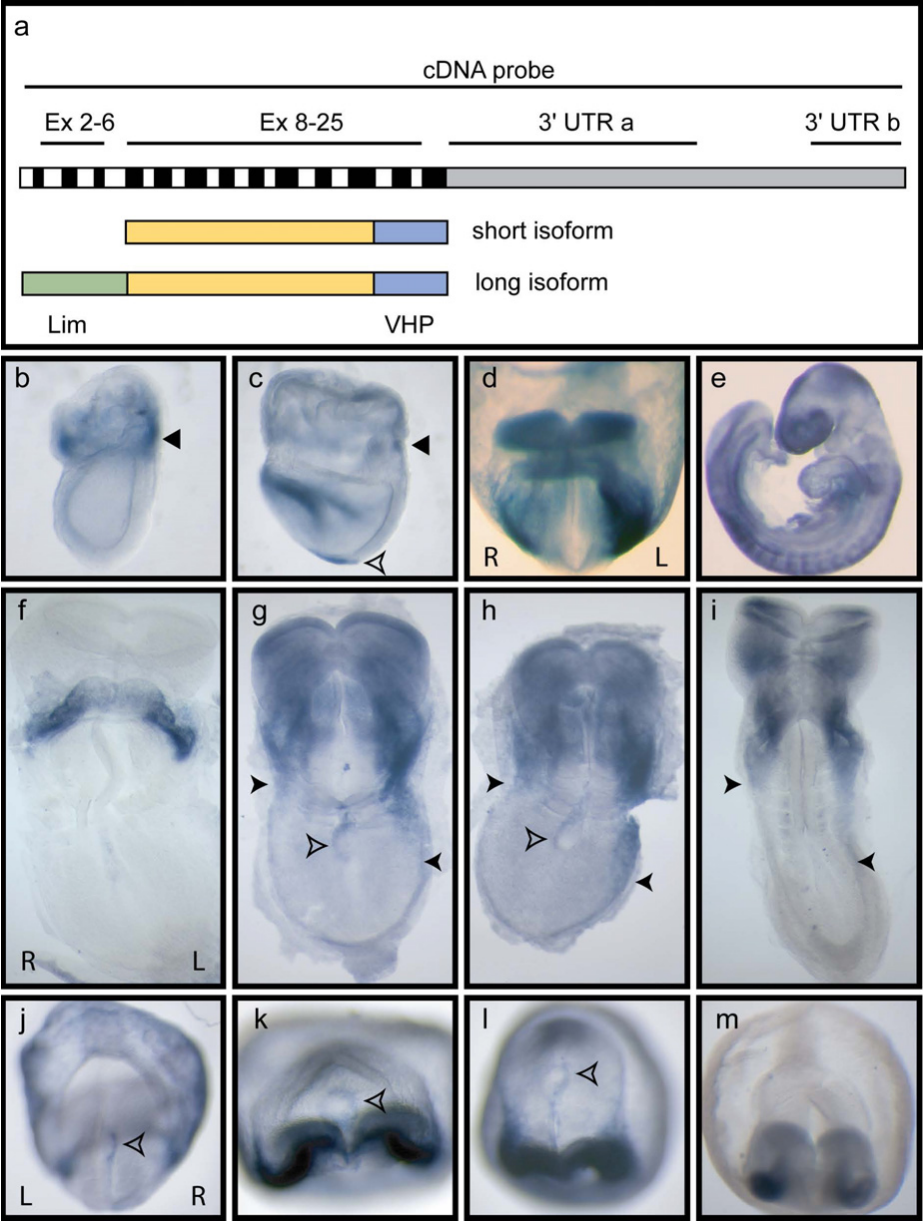
***Ablim1 *is a novel asymmetrically expressed locus**. (a) Diagram representing the *Ablim1 *transcript. The relative positions of the probes used for WISH analysis are marked at the top of the panel. The exon structure is indicated in the middle and the two major protein isoforms of *Ablim1 *are indicated at the bottom; the long isoform has 4 lim domains that the short form lacks. (b-m) Comparison of embryonic expression patterns of *Ablim1 *using probes to different mRNA regions. (b-e) Developmental expression of *Ablim1 *using the full length cDNA probe. (b) patchy yolk sac expression at 6.5 dpc, indicated by arrowhead, (c) a ring of expression in the yolk sac (arrowhead), head and node (open arrowhead) at 7.5 dpc, (d), symmetric expression in the head and yolk sac in addition to asymmetric expression is the lateral plate seen at 8.5 dpc, (e) expression in the heart, head and somites at 9.5 dpc. Expression at 8.5 dpc was assessed with Ex2-6 (f, j), Ex8-25 (g, k), 3'UTRa (h, l) and 3'UTRb (I, m). (f-i) flat mounted 8.5 dpc embryos dissected from their yolk sacs, visualised ventrally. (j-m) 8.5 dpc embryos within yolk sacs visualised from the posterior. The posterior most extent of LPM expression is indicated by a closed arrowhead and node expression is indicated by an open arrowhead. Panels (b), (c) and (e) are lateral views. In other panels Left (L) and Right (R) are as marked and are the same for each row of panels.

To identify genes asymmetrically expressed between left and right sides in the developing embryo we compared gene expression using the MRC Mouse Known Gene Oligo Array printed array slides (Mm_SGC_Av2), which identify 7455 known loci. Left and right lateral plate tissue was dissected from 3-6 somite embryos and pools from 4 embryos were used to prepare RNA. Following SMART PCR amplification, hybridisation and analysis were performed as described in the Materials and Methods. The results from 4 replicates were analysed, and lists ranking the apparent degree of left or right sided asymmetry were generated (see Additional file 1). The presence, at the top of the left-sided list of the known left specific gene, *Pitx2*, demonstrated the validity of the approach. Significantly, *Nodal*, though present on the array, was not identified; *Lefty2 *was not present on the array. We further analysed expression of 7 loci from the left-sided and 6 from the right sided list by RNA wholemount in situ hybridisation (WISH) on 8.5 dpc embryos. Of the 13 loci examined, only one, *Ablim1*, showed apparent L-R asymmetry of expression (Fig. 1 and data not shown). Expression in the left LPM was clearly stronger and more extensive than in the right, while a second asymmetric domain was visible at the node. Initial analysis of a few embryos showed expression predominantly on the right hand side of the node.

Performing WISH using the full cDNA, we examined *Ablim1 *expression in a developmental series of embryos, from 6.5 dpc to 9.5 dpc (Fig. 1b-e). Expression was detected in the yolk sac of 6.5 dpc embryos, initially as patches (Fig. 1b) that became a distinct ring of expression by 7.5 dpc (Fig. 1c), consistent with it marking the prospective blood islands. At 7.5 dpc, expression was also seen in the developing head folds (Fig. 1c), an expression pattern maintained through 9.5 dpc (Fig. 1d, e). *Ablim1 *expression in the node was seen from 7.5 dpc (Fig. 1c). By 8.5 dpc L-R symmetric expression was evident in the developing heart. Strikingly, asymmetric expression was also seen in the LPM; stronger and more extensive on the left than the right (Fig. 1d). By 9.5 dpc expression was evident in the head, the somites and portions of the heart, but no L-R asymmetry was seen at this stage (Fig. 1e, data not shown).

### Two classes of *Ablim1 *transcript show asymmetric expression in the lateral plate and the node respectively

Analysis of the published data [13] and ESTs http://www.ensembl.org demonstrates the existence of multiple *Ablim1 *transcripts, exhibiting both alternative splicing and multiple alternative first exons. Northern blot and RT-PCR analysis confirmed the existence of *Ablim1 *transcripts in 7.5 and 8.5 dpc embryos (data not shown). While multiple protein isoforms of Ablim1 exist, they comprise two major classes; long forms containing lim domains and a villin head piece (VHP) and a short form lacking the lim domains [13] (Fig. 1a). We investigated the temporospatial distribution of the transcripts encoding these isoforms using WISH probes hybridising to different portions of the *Ablim1 *message; probes corresponding to the beginning (Ex 2-6), the common region (Ex 8-25) and the 3'UTR (3'UTRa and b; Fig. 1). The Ex 2-6 probe, encompassing the lim domains, revealed expression in the developing heart (Fig. 1f) as well as the right side of the node when WISH colour development was extended for longer times (Fig. 1j), but not in the lateral plate. The Ex 8-25 probe revealed asymmetric expression to the right side of the node and in the left lateral plate; symmetric expression was evident in the head and heart (Fig. 1g, k). The 3'UTRa probe showed similar expression to Ex8-25, although node expression was significantly weaker when compared to the other expression domains (Fig. 1h, l). The terminal 3'UTR probe, 3'UTRb, showed similar expression, but failed to detect node expression, arguing for a shorter 3'UTR in the node transcripts (Fig. 1i, m). Together these results argue that a transcript encoding a long isoform containing both lim domains and VHP is present in the node, while a shorter isoform (with no lim domains) is present in the left lateral plate; it is not possible to say from our WISH or RT-PCR analysis whether the short isoform is also present in the node.

### Asymmetric *Ablim1 *lateral plate expression mirrors *Nodal*

**Figure 2:**
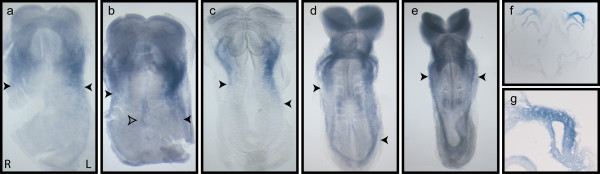
***Ablim1 *lateral plate asymmetry mirrors *Nodal***. *Ablim1 *is bilaterally symmetrical at 2 somites (a), becoming obviously asymmetric by 3 somites (b), when left lateral plate expression is stronger and more extensive than on the right. This WISH has been developed for longer allowing the asymmetric node expression to be visualised (open arrowhead). LPM asymmetry remains obvious at 5 somites (c) and some left LPM asymmetry is visible at 7 somites. Symmetrical expression in the more posterior somites is also evident. By 8 somites, all L-R asymmetry has been lost (e). Histology shows that Ablim1 is expressed throughout the lateral plate mesoderm (f, g). The posterior most extent of LPM expression is indicated by a closed arrowhead. In all panels the 3'UTRa probe has been used.

*Nodal*, often thought of as the master gene controlling left-sided gene expression, is expressed in the left but not right lateral plate from 3-6 somite stages [14,15]. If asymmetric *Ablim1 *expression is directly controlled by Nodal, it would be expected to exhibit similar temporospatial expression and not be expressed asymmetrically prior to asymmetric *Nodal *expression. We therefore examined the temporospatial expression of *Ablim1 *from the late headfold to the 10 somite stage by WISH (using the 3'UTRa probe). Up to and including the 2 somite stage, bilaterally symmetrical expression was seen in the anterior lateral plate, contiguous with expression in the heart (Fig. 2a). By 3 somites expression in the LPM became clearly asymmetric, with expression in the left lateral plate being both stronger and stretching noticeably further posteriorly than in the right (Fig. 2b). This asymmetric expression was highly evident at 5 somites, being particularly obvious when the WISH is developed for a short time (Fig. 2c). By 7 somites, after lateral plate *Nodal *expression has ceased, asymmetric left lateral plate expression of *Ablim1 *was strongly downregulated, although still evident at a low level in some embryos (Fig. 2d). By 8 somites, no sign of asymmetric lateral plate expression was evident (Fig. 2e). Analysis of sections revealed that lateral plate expression was present throughout the lateral plate mesoderm (Fig. 2f, g) similar to *Nodal*. These data are consistent with the hypothesis that *Nodal *activates *Ablim1*. As with our previous results, expression of *Ablim1 *in the node was apparent when the WISH colour development was left for longer periods of time (Fig. 1h, l, 2b).

### Left lateral plate *Ablim1 *expression occurs in the absence of detectable LPM *Nodal*

**Figure 3:**
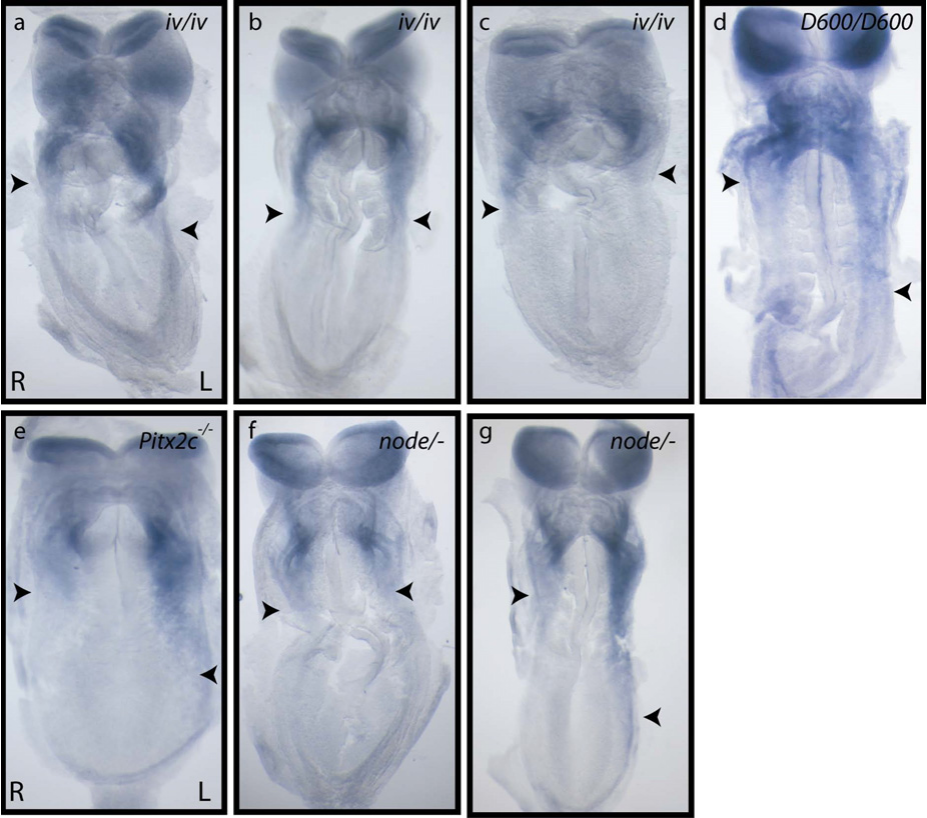
***Ablim1 *lateral plate asymmetry is maintained in the absence of *Nodal***. Expression, as assessed by WISH with the 3'UTRa probe. In *iv*/*iv *mutants (a-c), (a) left-sided, (b) bilateral and (c) right sided expression was detected. (d) Left-sided *Ablim1 *expression in a *Nodal^D600/D600 ^*(*D600*/*D600*) embryo. (e) Normal, left-sided expression of *Ablim1 *in a *Pitx2c^-/- ^*embryo. *Nodal^Δnode/- ^*(node/-) embryos show either loss of asymmetric *Ablim1 *expression (f) or left-sided *Ablim1 *expression (g). The posterior most extent of LPM expression is indicated by a closed arrowhead.

To further test the hypothesis that Nodal activates asymmetric *Ablim1 *expression, *Ablim1 *lateral plate expression was analysed in mutants with abnormal L-R patterning. *Dnahc11 *encodes a dynein heavy chain required for nodal cilia motility: the point mutant *Dnahc11^iv ^*(*iv*) results in immotile nodal cilia, absence of nodal flow, and randomisation of both situs and *Nodal *lateral plate expression [15-18]. When *Ablim1 *lateral plate expression was analysed in *iv *mutants, a mixture of expression patterns were seen (Table 1), similar to those previously reported for *Nodal *in the *iv *mutant [15], including left-sided (Fig. 3a), bilateral (Fig. 3b) and right sided (Fig. 3c) expression. These data show that *Ablim1 *asymmetry is downstream of nodal flow, similar to *Nodal *asymmetry and is consistent with *Ablim1 *asymmetry being downstream of *Nodal*. This hypothesis was further supported by analysis of *Shh *mutant embryos; *Shh *mutants do not express the Nodal antagonist *Lefty1 *in the midline, resulting in bilateral *Nodal *expression [19]. Consistent with a role for *Nodal *upstream of lateral plate *Ablim1 *expression, bilateral *Ablim1 *expression was seen in *Shh^-/- ^*embryos (data not shown and Table 1).

**Table 1:** LPM *Ablim1 *expression.

*Embryo*	*Left* *LPM*	*Left and Right* *LPM*	*Right* *LPM*	*No LPM* *Expression*
Wild Type	61 (80%)			15 (20%)
*iv*/*iv*	2 (11%)	8 (44%)	5 (28%)	3 (17%)
*Nodal^D600/D600^*	5 (56%)			4 (44%)
*Nodal^node/-^*	6 (24%)			19 (76%)
*Pitx2c^-/-^*	5 (100%)			
*Shh^-/-^*		4 (100%)		

We sought to analyse *Ablim1 *expression in embryos where *Nodal *LPM expression was strongly downregulated. *Nodal *contains an intronic enhancer (ASE) that is responsible for positive feedback regulation of its asymmetric expression in the left LPM. The *Nodal^D600 ^*allele lacks this ASE and consequently *Nodal^D600/D600 ^*embryos express extremely low levels of *Nodal *in the LPM [20,21]. This results in delayed and posteriorised activation of the Nodal target *Pitx2*, leading to L-R patterning defects. When *Ablim1 *expression was analysed in *Nodal^D600/D600 ^*embryos, ~50% of embryos showed asymmetric expression in left LPM (Fig. 3d; Table 1); the remainder were symmetrical, showing no LPM expression. However, the frequency at which no lateral plate *Ablim1 *expression was detected in *Nodal^D600/D600 ^*embryos was far higher than in wild type (Table 1). It is possible that the change in *Ablim1 *expression is a consequence of the delayed activation of the transcription factor *Pitx2 *in the *Nodal^D600/D600 ^*embryos. Therefore, we analyzed *Ablim1 *expression in *Pitx2 *mutants; *Pitx2c *is the isoform expressed asymmetrically in the left lateral plate mesoderm and *Pitx2c^-/- ^*embryos show defects in L-R patterning [22]. All 5 *Pitx2c^-/- ^*embryos analysed showed wild type *Ablim1 *expression (Table 1; Fig. 3e) demonstrating that *Pitx2c *expression is not required for *Ablim1 *asymmetry.

It seemed possible that the low level of LPM Nodal expressed in *Nodal^D600/D600 ^*embryos is borderline for activating asymmetric expression of *Ablim1 *in the left LPM and therefore leads to stochastic activation of the locus. To determine whether the 50% of *Nodal^D600/D600 ^*embryos that expressed asymmetric LPM *Ablim1 *resulted from low level *Nodal *expression, we analysed lateral plate *Ablim1 *expression in *Nodal^Δnode/- ^*embryos. The *Nodal^Δnode ^*allele lacks the enhancer that drives *Nodal *expression in the node [23]. *Nodal^Δnode/- ^*embryos have no, or occassionally a minimal ammount of *Nodal *expression at the node and no detectable *Nodal*, *Lefty2 *or *Pitx2 *in the LPM [23]. When *Ablim1 *expression was analysed in *Nodal^Δnode/- ^*mutant embryos, of 25 analysed, 6 (24%) showed clear asymmetric left LPM expression (Table 1, Fig. 3g). The remaining 19 (76%) showed no LPM *Ablim1 *expression on either the right or left sides (Table 1, Fig. 3f). These data clearly demonstrate that *Ablim1 *can be asymmetrically expressed in the LPM in the absence of detectable LPM Nodal signalling and therefore suggests that this asymmetric expression is regulated by signalling cues that are independent of the Nodal cascade. The reduced frequency of asymmetric *Ablim1 *expression in *Nodal^Δnode/- ^*embryos, however, suggests a role for *Nodal *in the robustness of *Ablim1 *lateral plate asymmetry. When we re-analysed these data with respect to the stage of development (Table 2), we saw no asymmetric LPM *Ablim1 *expression before 4 somites. Approximately 30% of 4-5 somite embryos showed asymmetric LPM *Ablim1 *expression. This increased to 50% when the 6-7 somite embryos were analysed. Together these data demonstrate that detectable LPM *Nodal *is required for early asymmetric left LPM *Ablim1 *expression, but, that in its absence a second asymmetric system can activate *Ablim1 *asymmetry.

**Table 2:** LPM *Ablim1 *expression in *Nodal^node/null ^*mutant embryos classified by somite stage*

*Nodal^node/null^*	*Asymmetry*	*No Asymmetry*
2-3 Somites	0 (0%)	9 (100%)
4-5 Somites	3 (30%)	7 (70%)
6-7 Somites	3 (50%)	3 (50%)

*(these are the same embryos analysed in Table 1)

### *Ablim1 *shows no functional ASE

In the lateral plate, Nodal's auto-activation of its own expression, as well as its activation of *Lefty2 *and *Pitx2 *expression, is mediated by binding of FoxH1 to asymmetric elements (ASEs). ASEs contain two or three FoxH1 binding sites (TGT G/T T/G ATT) within a 30-200 bp region. The random frequency of a pair of binding sites within such a region is once every 350 kb. When ASE-like sequences were sought around the mouse and human *Ablim1 *loci, 7 sequences were identified in mouse and 3 in humans (see Additional file 2), although position and overall sequence was not conserved. To address whether *Nodal *might be interacting with *Ablim1 *through these elements, the sequences plus 100 bp on either side were PCR amplified and cloned into luciferase reporter vectors. These were transfected into HepG2 cells together with *FoxH1 *and a constitutively active *Alk4 *construct. While a control fragment from the mouse *Pitx2 *ASE activated luciferase, as previously reported [24], all the *Ablim1 *derived fragments failed to activate expression above background levels, arguing that Nodal does not activate *Ablim1 *through FoxH1 binding to an ASE (data not shown).

### Highly dynamic *Ablim1 *node expression: a very early marker of asymmetry

**Figure 4:**
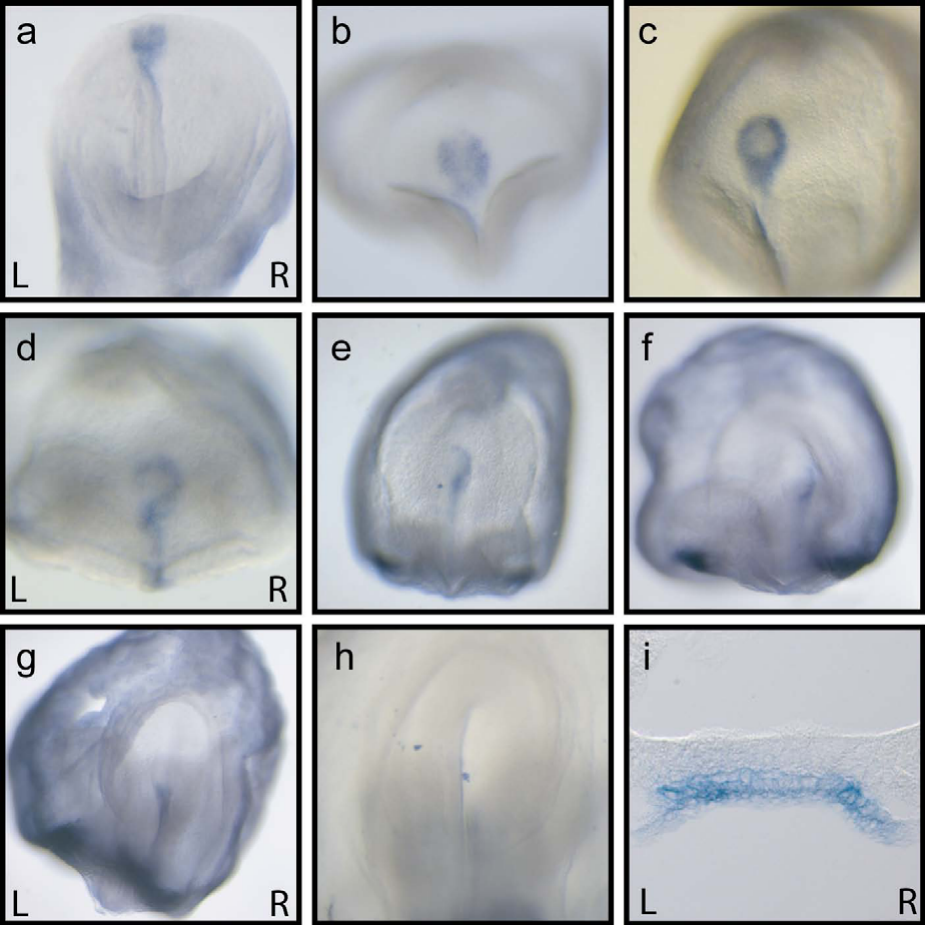
**A novel asymmetric expression pattern for *Ablim1 *at the node**. Temporal analysis of *Ablim1 *expression in the node, using the Ex2-6 probe. Initial expression across the node at the Early Headfold stage (a), becomes modified by the mid headfold stage (b) and by late headfold stages (c) describes a peri-nodal ring that shows stronger right than left-sided expression. By early somite stages (d) peri-nodal expression has been lost from the anteriormost left side of the node as well as being evident in the midline. (e) By 4 somites expression is restricted to the right side of the node. (f) At 5 somites node expression is restricted to the anteriormost right side and becomes restricted to the midline by 7 somites (g). (h) By 8 somites expression was absent form the node. (i) A section of a late headfold embryo (as in panel c) showing that expression in the node is restricted to the ventral layer of the node. Left (L) and Right (R) are as indicated on the panels.

When temporospatial *Ablim1 *expression was analysed in the node, utilising the Ex2-6 probe, transcript was detected from when the node is first patent (Fig. 4a, b). Intriguingly, this first expression is of a salt and pepper pattern stretching across the pit of the node. This resolved to give a ring surrounding the node by the mid-headfold stage (Fig. 4c); the first indication of asymmetry was evident at this stage, with a higher level of expression on the right than the left hand side of the node (Fig. 4c). During the next few hours of development, expression on the anterior left side of the node was lost, while expression was activated in the midline cells anterior to the node, resulting in a question mark-like expression pattern (Fig. 4d). By 4 somites, the remaining left-sided expression was lost, resulting in solely right-sided expression at the node (Fig. 4e). This abrogation of expression continued in a clockwise direction around the node (Fig. 4f), until by 7 somites peri-nodal expression was restricted to midline cells anterior to the node (Fig. 4g). By 8 somites midline expression has also been lost (Fig. 4h). Sections through these embryos reveal that expression in the node is restricted to the ventral layer (Fig. 4i).

### *Ablim1 *node expression is controlled by nodal flow and *Pkd2 *activity

**Figure 5:**
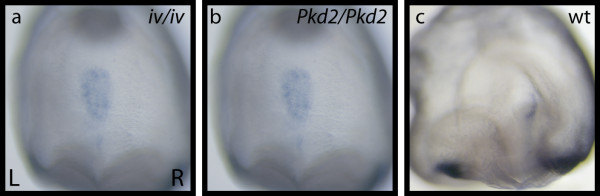
**Nodal flow and *Pkd2 *control *Ablim1 *expression at the node**. (a) *Ablim1 *expression in a 4 somite *Pkd2 mutant. (b) Ablim1 *expression in a 5 somite *iv *mutant. *(c) Ablim1 *expression in a 5 somite *wild type control embryo*. In both *iv *mutants, which lack nodal flow and *Pkd2 *mutants, *Ablim1 *expression was not detected in the usual peri-nodal ring; a patchy expression across the floor of the node was seen in a significant proportion of embryos, in striking contrast to wild type control embryos (c).

The early dynamic expression pattern of *Ablim1 *in the node correlates with the changes in nodal cilia motility and nodal flow described by Okada [18]. The loss of *Ablim1 *expression from the pit corresponds to the stage when local vortices form, while the loss of expression from the left side of the node correlates with the establishment of a strong leftwards flow. In conjunction with the very early asymmetry of *Ablim1*, this suggests that expression may be responding directly to nodal flow. To test this hypothesis we next examined *Ablim1 *node expression in *iv *mutants, where there is no nodal flow. Of 40 mutant embryos analysed, no asymmetry was detected at any stage of development. Indeed, the robust peri-nodal ring of expression was never detected. There was, however, a rise in the number of embryos where expression was not detected, from 12% in wt to 42% in *iv*/*iv *mutants (Table 3). Intriguingly, in the 58% of embryos where expression was detected, the pattern of expression was the same patchy expression seen in the very earliest wild type nodes (Fig. 5a). This pattern was maintained well through the period that leftwards laminar flow is normally detected and that strong asymmetry of *Ablim1 *is normally seen at the node. These data demonstrate that nodal flow is required for the upregulation of *Ablim1 *expression in the peri-nodal region and is involved in downregulation of *Ablim1 *expression in the pit of the node.

**Table 3:** Node *Ablim1 *expression.

*Embryo*	*Normal Node* *Expression*	*No Detectable Node* *Expression*	*"Patchy" Node* *Expression*
Wild Type	37 (88%)	5 (12%)	
*iv*/*iv*		17 (42%)	23 (58%)
*Pkd2*/LRM4		4 (44%)	5 (56%)
			

The two cilia hypothesis argues that nodal flow is directly detected by nodal cilia, through the activity of *Pkd2 *[7], resulting in a left-sided Ca2+ signal. To test whether *Ablim1 *may be responding to these asymmetric Ca2+ signals, we analysed expression in *Pkd2 *mutants. Surprisingly, very similar result were obtained to those for the *iv *mutant. From 9 mutants analysed, 4 (44%) showed no detectable expression, while 5 showed the same patchy expression we detected in the embryos lacking nodal flow (Fig. 5b). Once again, no L-R asymmetry was detectable. Together, these data demonstrate a requirement for both nodal flow and *Pkd2 *within the pit of the node for the loss of *Ablim1 *expression as well as for the establishment of asymmetric expression surrounding the node.

## Discussion

In this paper we describe the novel L-R asymmetric expression pattern of *Ablim1*, a gene that can be expressed independently of detectable *Nodal *in the left lateral plate mesoderm. A separate asymmetric expression domain in the embryonic node reveals both that *Ablim1 *expression is the earliest known marker of mammalian L-R asymmetry, and that flow (most likely mediated through Pkd2-dependent Ca2+ signalling) is directly affecting gene expression in the node, prior to any effects on asymmetry.

### A Nodal independent L-R asymmetric signal ("signal X")

**Figure 6:**
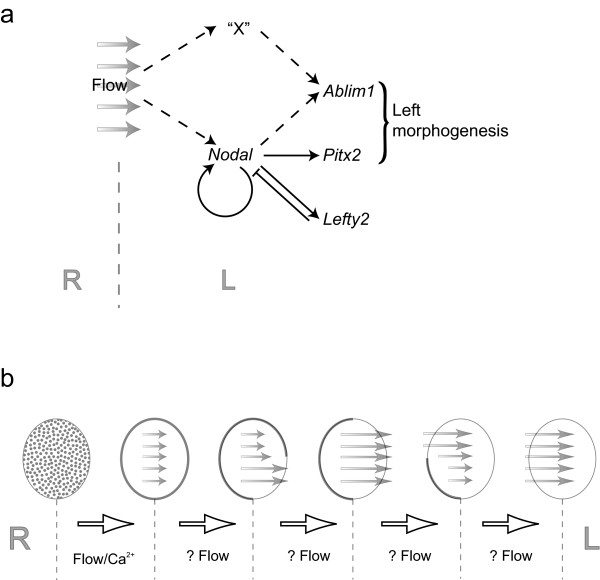
**A model explaining the control of *Ablim1 *expression in the node and the left LPM**. (a) Nodal flow and *Pkd2 *activate both the *Nodal*, *Pitx2 *pathway and a second pathway "X". Both pathways in turn activate *Ablim1 *in the left LPM. (b) In the node, the initial change of *Ablim1 *expression, from low level pan-nodal to a perinodal ring, require nodal flow and Pkd2 activity. Subsequent L-R asymmetry of perinodal ring expression may be controlled by nodal flow and its interaction with the remodelling of the node during development.

Work from many research groups over the past decade has established a generally accepted pathway underlying establishment of L-R patterning in mammals (reviewed [4]). The activation of the *Nodal *signalling cascade in the left LPM results in asymmetric, left-sided, expression of *Pitx2 *that is maintained into organogenesis (Fig. 6). In light of misexpression experiments in chick and Xenopus, *Pitx2 *has been argued to specify left sidedness [11,12]. Yet, while *Pitx2 *mutant mouse embryos demonstrate right pulmonary and atrial isomerism [25-28], the initial direction of embryonic turning and heart looping are *Pitx2*-independent [22]. In contrast, analysis of mice lacking or unable to respond to *Nodal *signalling in the lateral plate showed a randomised direction of heart looping [23,29]; the direction of embryonic turning was also randomised in *Cryptic *(MGI: *Cfc1*) mutants, but not reported for the *Nodal *mutants [23,29]. These data argue that heart looping and embryonic turning are controlled by *Nodal *expression, however, it is not possible to distinguish the role of Nodal at the node from its role in the lateral plate in these experiments. Our results clearly show *Ablim1 *asymmetry is independent of *Pitx2 *expression. More significantly, *Ablim1 *is capable of being asymmetrically expressed in the absence of detectable LPM *Nodal *signalling. Quite clearly, another L-R asymmetric signal is present in the embryo, that for the sake of discussion we shall refer to as "signal X".

### The control of *Ablim1 *LPM asymmetry

While it is clear from our analysis that *Ablim1 *can be asymmetrically expressed in the absence of detectable *Nodal *and *Pitx2*, asymmetric Nodal obviously does play a role. The strong reduction of lateral plate *Nodal *expression in *Nodal^D600/D600 ^*embryos results in ~50% of embryos with detectable asymmetric LPM *Ablim1 *expression, while the total removal of LPM *Nodal *expression in the *Nodal^Δnode/- ^*embryos reduces this level to 24% (Table 1). The level of LPM *Nodal *expression therefore directly affects asymmetric *Ablim1 *expression. This relationship is underlined when the temporal activation of *Ablim1 *is analysed; strong reduction of Nodal signalling is known to result in delayed target gene activation [20]. At early somite stages no Nodal-independent *Ablim1 *expression is seen, compared to expression in 30% of embryos by 4-5 somites and 50% by 6-7 somites (Table 2). It is therefore clear that two signals, a Nodal-dependent and the Nodal-independent "signal X" influence asymmetric lateral plate *Ablim1 *(Fig. 6A). While we have drawn "signal X" and Nodal both directly acting on *Ablim1*, we cannot rule out the possibility that "signal X" is in part regulated by Nodal. It is also possible that there is a temporal offset between Nodal signalling and "signal X", with Nodal acting first. The data neither argue for nor against these scenarios and in the absence of identifying and interfering with "signal X" it is impossible to distinguish between them.

### A left-sided "signal X"

A signal acting on the left side of the embryo may reflect either a left-sided activator or a right sided repressor of *Ablim1 *expression. Work from various groups has revealed additional lateral plate asymmetries in gene expression. BMP signalling, as assessed by Smad1 phosphorylation, is L-R asymmetric and this asymmetry is driven by asymmetric expression of the BMP antagonists chordin and noggin, which in turn are controlled by *Nodal *[30]. Therefore, it seems unlikely that BMP signalling lies upstream of *Ablim1 *asymmetry. Similarly, the *Nkx3-2 *(or *BapX1*) homeodomain locus shows right sided asymmetric expression, but again is thought to act downstream of *Nodal *[31]. In contrast, little is known about control of the right sided transcription factor Snail (*Snai1*), which temporally mirrors *Nodal *[32]. Conditional deletion leads to randomised embryonic turning and heart looping and bilateral activation of the *Nodal *signalling cascade, arguing that it normally inhibits right sided *Nodal *expression. It is therefore possible that *Snai1 *inhibits *Ablim1 *expression on the right side of the embryo. Indeed the role of the snail family of genes as transcriptional repressors is well documented [33]. A formal possibility exists that "signal X" in fact represents a very low level Nodal signal, below that capable of activating the known LPM targets of Nodal. While no *Nodal *expression is detected in the LPM, low level ectopic *Nodal *expression was reported in a few cells in the pit of the node in a small proportion of *Nodal^Δnode/- ^*embryos [23]. Such a localised signal would be expected to first activate expression close to the node, in a manner similar to that reported for initial *Nodal *activation [discussed in [4]. No such localised expression was evident for *Ablim1 *in *Nodal^Δnode/- ^*embryos (Fig. 3g and data not shown). Indeed, such putative low level *Nodal *signalling falls outside of the known activity of Nodal and it too would arguably constitue a novel asymmetric signal.

### Flow regulates early symmetrical *Ablim1 *expression at the node

Wild type *Ablim1 *expression in the node changes markedly at the mid-late headfold stage, from a low level broad pan-node expression to a robust peri-nodal ring (Fig. 4). This reflects two changes; upregulation in the crown cells surrounding the node and downregulation within the pit of the node. Developmentally this corresponds to when nodal cilia are driving vortical fluid motion [18], suggesting that cilia driven fluid flow plays a role in these expression changes. This is supported by the failure of *iv *and *Pkd2 *mutant embryos to express the peri-nodal ring of *Ablim1 *expression (Table 3). Moreover, almost 60% of these embryos maintain the same low level pan-node expression seen in early wild type embryos. We detected no expression in nodes of the remaining mutant embryos and argue that this reflects either failure to maintain low level pan-node expression over time and/or technical limitations in detecting low level gene expression by WISH. The role of nodal cilia motility in generating nodal flow and L-R asymmetry is so central to thinking that little consideration has been given to any earlier role for fluid flow. Our data, however, reveals an early and previously unrecognised function for nodal flow in modulating symmetrical gene expression within the node, separate from the role of nodal flow in L-R determination.

### *Ablim1 *is the earliest marker of L-R asymmetry in mouse

The first L-R asymmetry in *Ablim1 *expression is evident in the node by the late headfold stage (Fig. 4c), several hours before the 1-2 somite stage at which asymmetry is evident for the other known asymmetric loci *Nodal*, *Cerl2 *(MGI: *Dand5*) and *Lplunc1 *[14,15,34-36]. This argues that *Ablim1 *is responding to very early asymmetric signals. Nodal flow is argued to be the initial asymmetric signal in the mouse (reviewed [4]) and is clearly driving fluid flow leftwards by early somite stages [18]. However, the first *Ablim1 *asymmetry is evident at a stage when beads introduced into a node in vitro are carried leftwards only inefficiently and on average, hopping from vortex to vortex [18]. Whether such an inefficient flow could affect sensory cilia sufficiently to fulfil the requirements of the two cilia hypothesis is unclear. Presumably NVPs could be carried leftwards in a similar manner to the beads, although whether they would break efficiently in such a flow is uncertain.

By the early somite stages a completely novel and very distinctive asymmetry of *Ablim1 *expression becomes evident at the node. The peri-nodal ring "retreats" around the node in a clockwise direction between 3 and 7 somites. This asymmetry of expression is very different from that seen for other asymmetrically expressed loci at the node; these show bi-lateral expression flanking the node, with stronger expression on one side than the other.

While the control of *Ablim1 *node asymmetry is not addressed by this study, the speed of the changes in *Ablim1 *expression suggests that this is an active process. In chick an anti-clockwise migration of cells around the node underlies gene asymmetry [37,38]. However, in mice, cre-loxP based lineage analyses of both node crown and pit cells did not reveal such cell migration [23,39]. The role of flow in controlling earlier changes in *Ablim1 *expression makes flow a mechanism we must contemplate. Yet for flow to control *Ablim1 *asymmetry, the following objections must be taken into account. (1) How could leftwards flow initially affect just the anterior node? At early somite stages the anterior node is shallower than the posterior [40,41] and this may influence the ability of flow to impact on the crown cells in the anterior versus the posterior. (2) How does flow subsequently affect the posterior node? As the embryo grows the node remodels, becoming more even in depth between anterior and posterior. At the same time leftwards flow becomes laminar. A combination of these two events may then allow flow to also affect posterior left-sided node crown cells. (3) How does flow subsequently affect the right side of the node? In vivo, fluid flow within the node recycles being drawn downwards on the right hand side [42]. A combination of growth, node remodelling and perhaps temporal accumulation of signalling may allow the right side of the node to respond to the recycled flow as it is pulled back into the right hand side of the node (Fig. 6B).

While there is strong evidence for nodal flow in many vertebrates [43-45], earlier, pre-flow events have been demonstrated to influence L-R patterning in non-mammalian species. Vg1 can influence situs determination and its putative co-receptor, Syndecan-2, becomes asymmetrically phosphorylated in pre-flow Xenopus embryos [46-49]. Pharmacolgical experiments have implictaed H^+^K^+^ATPase, VATPase, Serotonin and 14-3-3 family member E in Xenopus situs determination [50-52]. In the resulting serotonin model, an electric field drives serotonin through gap junctions in Xenopus, resulting in higher right than left sided localisation [52-54]. The H^+^K^+^ATPase mRNA similarly becomes asymmetrically localised in Xenopus and perturbed expression disrupts L-R patterning in both Xenopus and chick [52]. Therefore the question must be raised as to whether such early mechanisms also exist in mammals and may be controlling *Ablim1 *asymmetry, either at the node or in the LPM.

That there is asymmetry of *Ablim1 *expression at both the node and the LPM bears comparison to the expression pattern of *Nodal*. *Nodal *asymmetry at the node slightly predates that in the LPM and is required for LPM expression in the mouse [23]. It has even been argued, in light of the ability of Nodal to autoactivate, that Nodal at the node might be carried to left LPM to activate expression there [55]. In striking contrast, *Ablim1 *asymmetry is on opposite sides in the node and LPM. So while there is *Ablim1 *asymmetry at the node when LPM asymmetry is first detected, the expression domains are on opposite sides of the embryo (Fig. 1g). Moreover, while Nodal is a signalling molecule, *Ablim1 *is a structural, cell autonomously acting protein. It is difficult to envisage how a cytoskeletal protein would be acting to repress its own expression across many cell diameters. More likely, the two expression domains are independently regulated. Indeed, the long isoform of the protein seen at the node but not detected in the LPM originates at an alternate first exon, consistent with different promoter enhancer combinations controlling the two expression domains.

### *Ablim1 *Function

Uniquely, for mammalian asymmetric genes, *Ablim1 *encodes a structural protein. As its name suggests, Ablim1 protein binds to actin and when first identified, the presence of lim domains led the authors to suggest that it might act as an adaptor protein, bringing other proteins to the actin cytoskeleton [56]. The homologue in *C. elegans*, *unc-115*, similarly binds actin [57], and when mutated leads to an uncoordinated phenotype and defects in axon guidance [58]. Expression of a dominant negative *Ablim1 *in chick embryos leads to similar axonal phenotypes [59]. Yang and Lundquist [60] further demonstrated that expression of *unc-115 *in mammalian fibroblasts led to the formation of peripheral actin conglomerations at the expense of stress fibres. One possible explanation that they suggest is that Ablim1 protein may have different roles at the cell membrane and in the cytoplasm, acting to abrogate stress fibre formation when cytoplasmic. This raises the possibility that asymmetric *Ablim1 *expression might prove permissive for asymmetric morphogenetic changes in the embryo. However, when an isoform specific deletion of *Ablim1 *was made, for the isoform seen in the eye, no defects were reported [13]. This deletion, however, seems unlikely to affect the expression that we have described. Indeed many additional alternative first exons are now annotated that were not evident to Lu and colleagues.

## Conclusion

We have identified *Ablim1 *as a L-R asymmetrically expressed LPM gene that also shows a highly novel asymmetric expression pattern in the node. Through study of *Ablim1*, we provide definitive evidence that in addition to the recognised *Nodal*-*Pitx2 *asymmetric pathway in the left LPM, a second LPM *Nodal*-independent pathway must exist.

In the node we reveal a previously unrealised role for flow and *Pkd2 *in the control of early symmetrical *Ablim1 *expression within the node. This provides a novel, expression based readout of nodal flow. It seems reasonable to speculate that other genes expressed within the node may also be modulated by fluid flow.

*Ablim1 *expression within the early node becomes asymmetric at the head fold stage, several hours previous to other asymmetrically expressed loci. This is the earliest marker of L-R asymmetry in the mouse. Subsequent asymmetric node expression proceeds in an entirely novel pattern, retreating around the node. While we do not understand how this is controlled, future study of this seems likely to shed light on the mechanisms of L-R patterning.

*Ablim1 *is a candidate for processes controlling L-R morphogenesis and identity and future study of mutants will reveal it role in L-R patterning.

## Methods

### Mice

To collect staged embryos, mating was assessed by monitoring for copulation plugs. The day of plug was designated day 0.5. Wild type embryos were (C3H/HeH × 101/H)F1s. Mutant lines were: *iv*- *Dnahc11^iv ^*[61,62]; *Pkd2^lrm4 ^*[63]; *Nodal^D600^*, *Nodal^tm2Rob ^*[20]; *Nodal^- ^*is the Nodal-LacZ allele *Nodal^tm1Rob ^*[14]; *Nodal^Δnode ^*is the Nodal node del, *Nodal^tm3Rob ^*[23]; *Pitx2c *null, *Pitx2^tm3.1Jfm ^*[22]; *Shh*, *Shh^tm1Chg ^*[64].

All animals were used in accordance with UK Home Office regulations.

### Microarrays

Tissue for micro-array analysis was dissected in cooled PBS, then snap frozen in liquid nitrogen. RNA was produced by Qiagen RNeasy mini kit and quality assessed by Agilent 2100 Bioanalyzer. Reverse transcription and amplification were conducted according to the SMART mRNA Amplification Kit (Clontech). The resulting samples were labelled with Cy3 and Cy5 and used to hybridise MRC Mouse Known Gene Oligo Array printed array slides (Mm_SGC_Av2), identifying 7455 known genes, according to standard protocols. Results from 4 experimental repeats were analysed using GeneSpring (Agilent Technologies). Genes were ranked for differences in left versus right sided expression. The data from these experiments has been submitted to ArrayExpress, reference E-MEXP-2277.

### Molecular biology

The following IMAGE clones (IC) were used to produce anti-sense WISH probes: IC1-3995027 *Oaz1*, IC2-4457726 *Agtrl1*, IC3-4935411 *Ablim1*, IC4-5042041 *Tssc3*, IC5-5123607 *F10*, IC6-5291579 *Fkbpla*, IC7-5329923 *Cts1*, IC8-5716506 *Plk*, IC9-6335891 *Hmgi*, IC10-6390454 *Aqp3*, IC11-3963483 *Tpm1*, IC12-5715646 *tuba1*, IC13-5685601 *Hyal1*.

IC3 represents a full length *Ablim1 *clone. Clones portions of *Ablim1 *sequence, exons 2-6, 8-25 and 3'UTR sequences, were produced by restriction digest or PCR amplification and cloned into pBluescript2. Antisense RNA probes were produced and used for wholemount in situ hybridisation, according to standard protocols.

Putative ASE sequences were amplified by PCR from BALB/c mouse and commercial human DNA, TA cloned into pGL3-Promoter vector (Promega). The sequence cloned into pGL3 was confirmed by sequencing. Luciferase assays were carried out as previously described [65].

## Abbreviations

ASE: asymmetric element; EST: expressed sequence tag; LPM: Lateral plate mesoderm; L-R: Left-Right; UTR: untranslated region; VHP: villin headpiece; WISH: wholemount in situ hybridisation.

## Authors' contributions

JS carried out the majority of dissections, genotyping and molecular genetic analysis. AE carried out additional practical work and contributed to experimental design. HH and PU performed and analysed the microarray experiment. JB and SB planned and performed the luciferase analysis. NB provided genotyped mutant embryos for analysis. DN conceived, designed and supervised the project and wrote the manuscript. All authors read and approved the final manuscript.

## Supplementary Material

Additional file 1 **L-R micro array data**. The top asymmetric expressing genes as indicated by the micro-array analysis. Data from 4 experiments has been averaged and genes ranked for stronger left (top) or right (bottom) sided expression. Genes subsequently analysed by in situ are highlighted. Click here for file

Additional file 2 **ASE containing sequences around *Ablim1***. Sequences containing two or more FoxH1 binding sites (TGT G/T T/G ATT) within a 30-200 bp region, in and surrounding the mouse and human *Ablim1 *loci. Click here for file
