# Supplementary material for: Analysis of the asymmetrically expressed Ablim1 locus reveals existence of a lateral plate Nodal-independent left sided signal and an early, left-right independent role for nodal flow
Source: BMC Dev Biol. 2010 May 20;10:54. doi: 10.1186/1471-213X-10-54 (PMC2885315; doi:10.1186/1471-213X-10-54)
Supplement: Additional file 2 — ASE containing sequences around Ablim1. Sequences containing two or more FoxH1 binding sites (TGT G/T T/G ATT) within a 30-200 bp region, in and surrounding the mouse and human Ablim1 loci. [file 1471-213X-10-54-S2.DOC]

Mouse

>19:[25260,25335]

AATACACA CTTTCCCCTGCCTAGGACACGCTTCTCCCCTGCCTAGGATGCTGACCTGTGAGTTTTCCT TGTGTATT

>19:[103446,103577]

AATACACA AGGCCAAGGTGGAGCTGAAGCTCTGAAGCATGGGAGGGGGCTGCTGCAGTCAGGATGCCCAGCAGAGGAAACAGGCTGGGAGTGGCCAGGCATGGATGTCCAGCCATCCTCGACCA TGTGGATT

>19:[207412,207471]

AATACACA TTTGCTCATAGAAAATTTCAAAAGCACTAGAAAAATTTTTGAGA AATAAACA

>19:[210814,210683]

AATCCACA TGCCCTAGGATTGCAATTGTACACACCACATGCACTGGGATTGTGAGTGTGCACTCACTGCACATGCACTGGGATTGCAAGTGTGCACTGCACATGCACTGGGATTGAAAGTGTAC AATCCACA

>19:[208620,208513]

AATACACA CCACCACAACTACATGCAACCATGACCATGGCTCACACAACCATGACCACAGCACATACATCCACAACCATGGCACACAACTCCATAACCAC AATACACA

>19:[103577,103446]

AATCCACA TGGTCGAGGATGGCTGGACATCCATGCCTGGCCACTCCCAGCCTGTTTCCTCTGCTGGGCATCCTGACTGCAGCAGCCCCCTCCCATGCTTCAGAGCTTCAGCTCCACCTTGGCCT TGTGTATT

>19:[25335,25260]

AATACACA AGGAAAACTCACAGGTCAGCATCCTAGGCAGGGGAGAAGCGTGTCCTAGGCAGGGGAAAG TGTGTATT

Human

>10:[189847,189942]

AATCAACA CCAAACTGGTAGCTTCTTGGATGGCAGGTCTTTTTCTGCTTAGAGGTCTCCCAAGTGTTGTTGCTTAATTCTCACATCTC AATACACA

>10:[280816,280900]

AATAAACA CCTAGAAGACACATACCTGCCCAAGAAATGTTACTACATCATTGTCTCTCTCCTGCACTGTTCACTCAG TGTGTATT

>10:[280900,280816]

AATACACA CTGAGTGAACAGTGCAGGAGAGAGACAATGATGTAGTAACATTTCTTGGGCAGGTATGTGTCTTCTAGG TGTTTATT
